# Three-dimensional arterial spin labeling-guided dose painting radiotherapy for non-enhancing low-grade gliomas

**DOI:** 10.1007/s11604-022-01357-z

**Published:** 2022-11-07

**Authors:** Zihong Zhu, Guanzhong Gong, Lizhen Wang, Ya Su, Jie Lu, Yong Yin

**Affiliations:** 1 Department of Oncology, Affiliated Hospital of Southwest Medical University, No.25 Taiping Street, Jiangyang District, Luzhou, 646000 Sichuan China; 2 Department of Radiation Oncology Physics and Technology, Shandong Cancer Hospital and Institute, Shandong First Medical University and Shandong Academy of Medical Sciences, No.440 Jiyan Road, Huaiyin District, Jinan, 250117 Shandong China

**Keywords:** Three-dimensional arterial spin labeling, Low-grade gliomas, Non-enhancing, Dose painting, Radiotherapy

## Abstract

**Purpose:**

To investigate the feasibility and dosimetric characteristics of dose painting for non-enhancing low-grade gliomas (NE-LGGs) guided by three-dimensional arterial spin labeling (3D-ASL).

**Materials and methods:**

Eighteen patients with NE-LGGs were enrolled. 3D-ASL, T2 fluid-attenuated inversion recovery (T2 Flair) and contrast-enhanced T1-weighted magnetic resonance images were obtained. The gross tumor volume (GTV) was delineated on the T2 Flair. The hyper-perfusion region of the GTV (GTV-ASL) was determined by 3D-ASL, and the GTV-SUB was obtained by subtracting the GTV-ASL from the GTV. The clinical target volume (CTV) was created by iso-tropically expanding the GTV by 1 cm. The planning target volume (PTV), PTV-ASL were obtained by expanding the external margins of the CTV, GTV-ASL, respectively. PTV-SUB was generated by subtracting PTV-ASL from PTV. Three plans were generated for each patient: a conventional plan (plan 1) without dose escalation delivering 95–110% of 45–60 Gy in 1.8–2 Gy fractions to the PTV and two dose-painting plans (plan 2 and plan 3) with dose escalating by 10–20% (range, 50–72 Gy) to the PTV-ASL based on plan 1. The plan 3 was obtained from plan 2 without the maximum dose constraint. The dosimetric differences among the three plans were compared.

**Results:**

The volume ratio of the PTV-ASL to the PTV was (23.49 ± 11.94)% (*Z* = − 3.724, *P* = 0.000). Compared with plan 1, D_2%_, D_98%_ and D_mean_ of PTV-ASL increased by 14.67%,16.17% and 14.31% in plan2 and 19.84%,15.52% and 14.27% in plan3, respectively (*P* < 0.05); the D_2%_ of the PTV and PTV-SUB increased by 11.89% and 8.34% in plan 2, 15.89% and 8.49% in plan 3, respectively (*P* < 0.05). The PTV coverages were comparable among the three plans (*P* > 0.05). In plan 2 and plan 3, the conformity indexes decreased by 18.60% and 12.79%; while the homogeneity index increased by 1.43 and 2 times (*P* < 0.05). Compared with plan 1, the D_0.1 cc_ of brain stem and D_max_ of optic chiasma were slightly increased in plan 2 and plan 3, and the absolute doses met the dose constraint. The doses of the other organs at risk (OARs) were similar among the three plans (*P* > 0.05).

**Conclusion:**

The dose delivered to hyper-perfusion volume derived from 3D-ASL can increased by 10–20% while respecting the constraints to the OARs for NE-LGGs, which provides a basis for future individualized and precise radiotherapy, especially if the contrast agent cannot be injected or when contrast enhancement is uncertain.

## Introduction

Non-enhancing low-grade gliomas (NE-LGGs) are a special type of LGGs. Grade II Astrocytoma was the most common histological type. The vast majority of non-enhancing gliomas are LGGs, and LGGs enhanced as commonly as they lacked enhancement [1, 2]. These NE-LGGs was not enhanced on conventional enhanced computed tomography (CT) or magnetic resonance imaging (MRI). Their blood vessel wall composition is similar to that of brain-healthy tissue, with a complete blood–brain barrier. Macromolecules used for tumor enhancement cannot pass through the blood–brain barrier to enhance these lesions. Therefore, the lesions show low density on enhanced CT and T1 hypo-intensity and T2 hyper-intensity on enhanced MRI (the manifestation of edema) [3, 4]. Radiotherapy is one of the effective treatment methods for NE-LGGs, which is usually the first option for patients who are unable to operate.

Most LGGs recurrence after radiotherapy predominantly occurs in the radiation field in conventional radiotherapy, which is significantly associated with insufficient doses to the tumor target region [5, 6]. Based on conventional anatomical MR images for homogeneous dose boosting, the dose of healthy tissue increases simultaneously, and the occurrence of radiation damage is inevitable. In contrast, the dose-painting radiotherapy guided by functional images, such as functional MR and positron-emission tomography (PET), which prescripts heterogeneous dose distribution to the target volume, is expected to achieve a safe dose boost on the premise of ensuring the safety and the accuracy of dose transmission. In a prospective phase II randomized clinical trial, it investigated the region of hyper-perfused/hyper-cellular (TV_HCV_/TV_CBV_) > 1 cm^3^ in high b-value diffusion-weighted MRI and dynamic contrast-enhanced perfusion (DCE) MRI for glioblastoma with dose boosting (75 Gy/30 f) and found that patients treated to the combined hyper-cellular/hyper-perfused tumor volume had significantly improved 12-month overall survival (OS) rate (92%; 95% CI 78–100%) compared with historical control (*P* = 0.03). However, there was no significant difference in 12-month OS between patients boosted to the hyper-cellular tumor volume alone vs historical control (*P* = 0.9) with favorable outcomes for neurocognitive function, symptom burden, and quality of life [7].

At present, the abnormal hyper-intensity (edema area) on T2 fluid-attenuated inversion recovery (T2 Flair) is usually used as the standard for delineating gross tumor volume (GTV), which can ensure the coverage of radiotherapy dose to tumor, but it is challenge to increase the radiotherapy dose due to the large irradiation range. Although the tumor boundary of NE-LGGs is difficult to identify in conventional imaging, the blood flow and metabolism of the tumor are still significantly different from those of the brain tissue in the edema area in biology [8]. Quantifying this difference through functional imaging can clarify the distribution of tumor cell enrichment areas, which could guide the delineation of target volume during radiotherapy. Commonly functional imaging, including dynamic susceptibility contrast MRI (DSC), DCE or PET, requires intravenous administration of a contrast agent and is therefore somewhat limited.

As a non-invasive blood perfusion imaging technology, three-dimensional arterial spin labeling (3D-ASL) generates an image by magnetically labeling water molecules as an endogenous tracer. It does not require the destruction of the blood–brain barrier and can reflect microscopic changes in tissue blood perfusion and micro-vessel density [9, 10]. 3D-ASL perfusion imaging can effectively distinguish intracranial tumors from non-neoplastic lesions more sensitive than conventional MR sequences for the detection of tumor progression or recurrence [11–13]. The tumor cerebral blood flow (CBF) value is significantly negatively correlated with survival [14]. ASL has been used to guide stereotactic biopsy and operation for glioma. Jin et al. [15] showed that compared with conventional MRI, combined magnetic resonance spectroscopy (MRS) and ASL improved the accuracy of target selection for the stereotactic biopsy of intracranial tumors, especially three cases each of low-enhancing and non-enhancing gliomas. Lindner et al. [16] concluded that intraoperative ASL was a feasible, reproducible, and reliable tool to map CBF in brain tumors and seemed to give beneficial information compared to conventional intraoperative MRI in partial resection.

3D-ASL has been widely used in the diagnosis, grading and efficacy evaluation of gliomas [17, 18]. However, there are few reports on its application in guiding the radiotherapy planning of NE-LGGs. In this study, we investigated whether dose painting for NE-LGGs can achieve dose escalation to hyper-perfusion region derived from 3D-ASL without increasing the dose to cranial organs at risk (OARs).

## Materials and methods

### Patients

This study enrolled 18 patients between December 2018 and May 2021. Eligibility criteria: (1) Low-grade glioma confirmed by histopathology and molecular characteristics; (2) no enhancement on enhanced CT and MR; (4) 3D-ASL scanning prior to treatment. Patients, including 12 (67%) males and 6 (33%) females, with an average age of 42 ± 21 years (5 to 74 years). Radiological studies performed on the patients were accessed by two neuroradiologists from Picture Archiving and Communication Systems (PACS). WHO grading was reviewed by three neuropathologists according to the fifth edition of the WHO Classification of Tumors of the Central Nervous System (WHO CNS5) [19]. Patient’s characteristics are presented in Table 1.

**Table 1:** Clinical characteristics of the patients

Number of patients	18
Gender	
Male	12 (67%)
Female	6 (33%)
Age, mean ± standard deviation	42 ± 21
Male, mean ± standard deviation	43 ± 21
Female, mean ± standard deviation	40 ± 21
Age phases	
Adults (> 18 years)	15 (83%)
Children (< 18 years)	3 (17%)
Tumor location	
Unilateral	7 (39%)
Midline	8 (44%)
Bilateral (diffuse distribution)	3 (17%)
Type of histopathology	
Astrocytoma	18 (100%)
WHO classification*	
Grade II	18 (100%)
IDH mutation status	
Mutant	1 (6%)
Wild type	13 (72%)
Unclear (NOS)	4 (22%)
1p/19q deletion status	
Co-deletion	0 (0%)
No deficiencies	4 (22%)
Unclear	14 (78%)
Concurrent temozolomide chemoradiotherapy	
Yes	14 (78%)
No	4 (22%)
Median observation time, median (range)	11.5 months (1 months –29 months)

*Grading of all enrolled patients according to the fifth edition of WHO classification of tumors of the central nervous system. IDH, iso-citrate dehydrogenase

### CT and MRI images acquisition

#### CT

A CT scanner (Brilliance Big Bore, Philips, Netherlands) with a slice thickness of 3 mm and no inter-slice gap width was used, scanning from the foramen magnum to the end of the skull in a caudo-cranial direction.

#### MRI

All MR images (T2 Flair, 3D-ASL and contrast-enhanced T1-weighted (CE-T1W)) were acquired with the same 3.0 T MR scanner (Discovery 750 W, GE Healthcare, USA) using a standard 8-channel cranial coil. For CE-T1W images, gadopentetate dimeglumine was injected at doses standardized by patient’s body weight (0.2 mL/kg) at 2 mL/s, and the scan was started 3–5 min after the injection. The common scan parameters for all sequences are shown in Table 2.

**Table 2:** MRI simulated parameters for each sequence

Parameters	T2/FLAIR	3D-ASL	CE-T1WI
TR (ms)	11,000	5160	8.5
TE (ms)	120	11.5	3.2
Layer thickness (mm)	3	3	3
FOV (cm)	26	25.6	25.6
Matrix	320 × 320	512 × 512	256 × 256

*T2 FLAIR* T2 fluid-attenuated inversion recovery, *CE-T1WI* contrast-enhanced T1-weighted imaging, *3D-ASL* three-dimensional arterial spin labeling, *TR* repetition time, *TE* echo time, *FOV* field of view

### 3D-ASL data acquisition and post-processing

Setting a marking plane on the neck in advance, labeling with pseudo-continuity, image acquisition with single-shot gradient-echo echo planar imaging (EPI), labeling duration = 1500 ms, post spin labeling delay (PLD) = 2025 ms, number of excitation (NEX) = 3, forty pairs of control/label images were acquired and averaged. The scan duration was 5:27. For measurement of the magnetization of arterial blood and also for segmentation purposes, an M0 calibration image was acquired separately with the same geometry and the same imaging parameters as the ASL without labeling. Background suppression technology was used in the process of image acquisition.

The raw 3D-ASL data were transferred to the GE Advantage Workstation 4.7 and post-processed in the FuncTool environment. The motion correction, temporal and spatial filtering, and partial volume effect correction were performed. Subsequently, the CBF values were calculated by intensity normalization. A quantitative CBF map was generated based on the following Equation [20]:

$$\mathrm{CBF}=\frac{6000\bullet \lambda \bullet ({\mathrm{SI}}_{\mathrm{control}}{-\mathrm{SI}}_{\mathrm{label}})\bullet {\mathrm{e}}^{\frac{\mathrm{PLD}}{{T}_{1,\mathrm{blood}}}}}{2\bullet \alpha \bullet {T}_{1,\mathrm{blood}}\bullet {\mathrm{SI}}_{\mathrm{PD}}\bullet (1-{\mathrm{e}}^{-\frac{\tau }{{T}_{1,\mathrm{blood}}}})}\left[\mathrm{ml}/100\mathrm{g}/\mathrm{min}\right]$$

where *λ* is the brain/blood partition coefficient in ml/g, SI_control_ and SI_label_ are the time-averaged signal intensities in the control and label images, respectively, *T*_1,blood_ is the longitudinal relaxation time of blood in seconds, α is the labeling efficiency, SI_PD_ is the signal intensity of a proton density-weighted image, τ is the label duration, and PLD is the post-labeling delay. The parameters used in the present study were: *λ* = 0.9, *T*_1,blood_ = 1650 ms, *α* = 0.85, *τ* = 1500 ms and PLD = 2025 ms. The SI_PD_ was derived from the echo planar imaging M0 images. The factor of 6000 converts the units from ml/g/s to ml/100 g/ min, which is customary in the physiological literature.

### Target volume delineation

All MRI images were imported into MIM Maestro software (6.8.8, USA). GTV-ASL was defined as the high-perfusion volume with a relative CBF value greater than 1.4 in 3D-ASL images, where the relative CBF value was the CBF ratio of tumor to contralateral mirror gray matter (the unilateral cerebral hemisphere) or normal gray matter of the left insula lobe (midline or widely diffuse distribution), as previously published [21]. An automatic threshold method was used to generate GTV-ASL, and a senior radiologist later reviewed and modified the report as needed. Axial T2 Flair sequences were used to contour the hyper-intense abnormality as the GTV. The GTV-SUB was obtained by subtracting the GTV-ASL from the GTV.

**Fig. 1:**
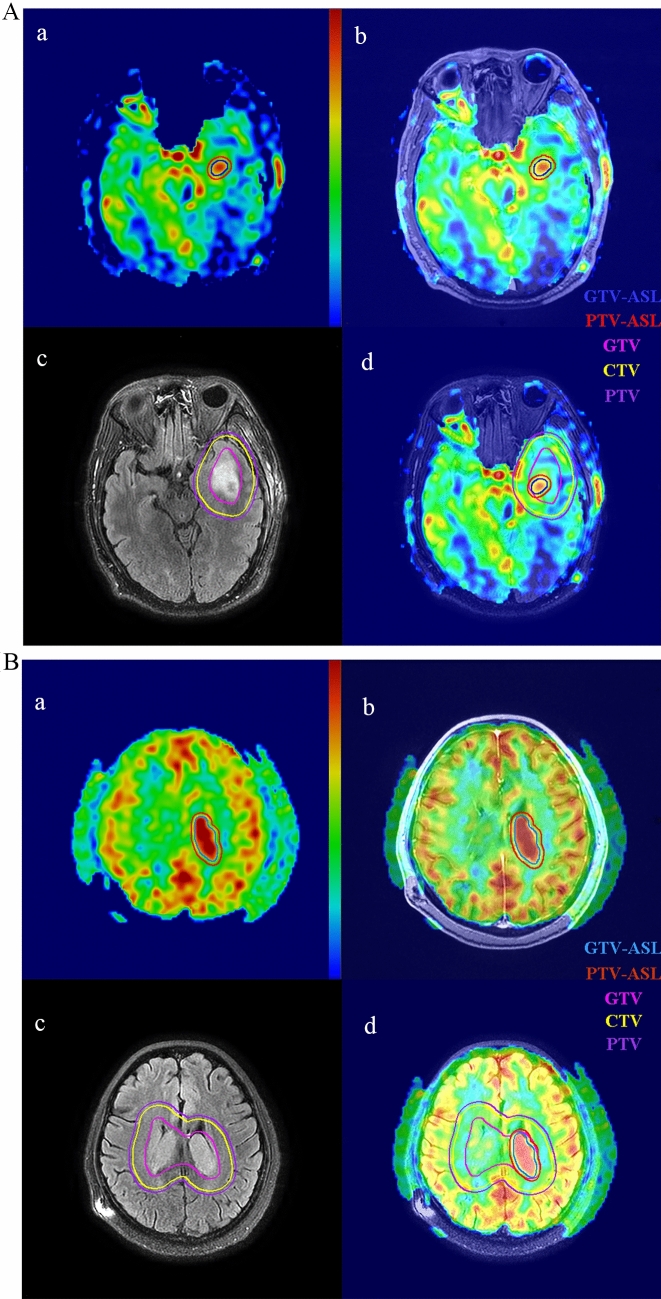
**A**, **B** Target volumes shown on **a** three-dimensional arterial spin labeling (3D-ASL), **b** fusion image of contrast-enhanced T1-weighted imaging (CE-T1WI) and 3D-ASL, **c** T2 fluid-attenuated inversion recovery (T2 Flair), and **d** fusion image of T2 Flair and 3D-ASL for two patients with non-enhancing low-grade gliomas (NE-LGGs) located in the left temporal lobe (**A**) and in the thalamus (**B**). GTV-ASL, the high-perfusion region on 3D-ASL; GTV, the hyper-intense abnormality on T2 Flair (conventional GTV); *CTV* clinical target volume, *PTV* the planning target volume

Clinical target volumes (CTV) were created by iso-tropically expanding the GTV by 1 cm. The planning target volume (PTV) and PTV-ASL were generated by adding a 0.5 and 0.3 cm margin to CTV and GTV-ASL, respectively. The PTV-SUB was obtained by subtracting the PTV-ASL from the PTV. The PTV-ASL was used to generate simultaneous integrated boost (SIB) volume. OARs used for treatment planning were: eyeballs, lenses, optic nerves, optic chiasma and brain stem. Figure 1A, B shows the target volumes delineated for two patients with NE-LGGs.

### Radiotherapy planning

All treatment plans were designed in the Eclipse (Version 15.6, Varian, USA) treatment planning system using static intensity-modulated radiotherapy (IMRT). Three plans were generated for each patient: a conventional plan (plan 1) without dose escalation delivering 95–110% of 45–60 Gy in 1.8–2 Gy fractions to the PTV and two dose-painted plans (plan 2 and plan 3) with dose escalating by 10–20% (range, 50–72 Gy) to the PTV-ASL based on plan 1. Plan 1 and plan 2 were generated with the maximum dose constraint of 110% of the prescription dose, and the plan 3 was obtained from plan 2 without the maximum dose constraint. The prescription covered 95% of the target volume. The prescription dose ultimately depended on the location of tumor. Seven patients with unilateral hemispheric tumors were prescribed 60 Gy in plan 1, and the dose of PTV-ASL increased by 20% (72 Gy) in plans 2 and 3. For other patients, the prescription doses were relatively limited because the tumors were located in important functional areas such as the midline of the brain or widely distributed. The prescription doses were 45–54 Gy in plan 1, and the dose of PTV-ASL increased by 10–20% (50–60 Gy) in plan 2 and plan 3.

The dose normalizations were set to the average dose of PTV in plan 1 and PTV-ASL in plans 2 and plan 3. The field angles were constant for all three treatment plans. Dose calculation was performed in anisotropic analytical algorithm optimization mode (AAA, version 15.512) using 6 MV X-rays. The calculated grid size was 2.5 mm × 2.5 mm.

The doses of OARs were restricted as following: brain stem D_0.1 cc_ (the dose to 0.1 cc target volume) ≤ 54 Gy, eyeballs D_max_ (the maximum dose) < 45 Gy, lens D_max_ < 10 Gy, and optic nerves and optic chiasm D_max_ ≤ 55 Gy.

### Evaluation of treatment plans

**Fig. 2:**
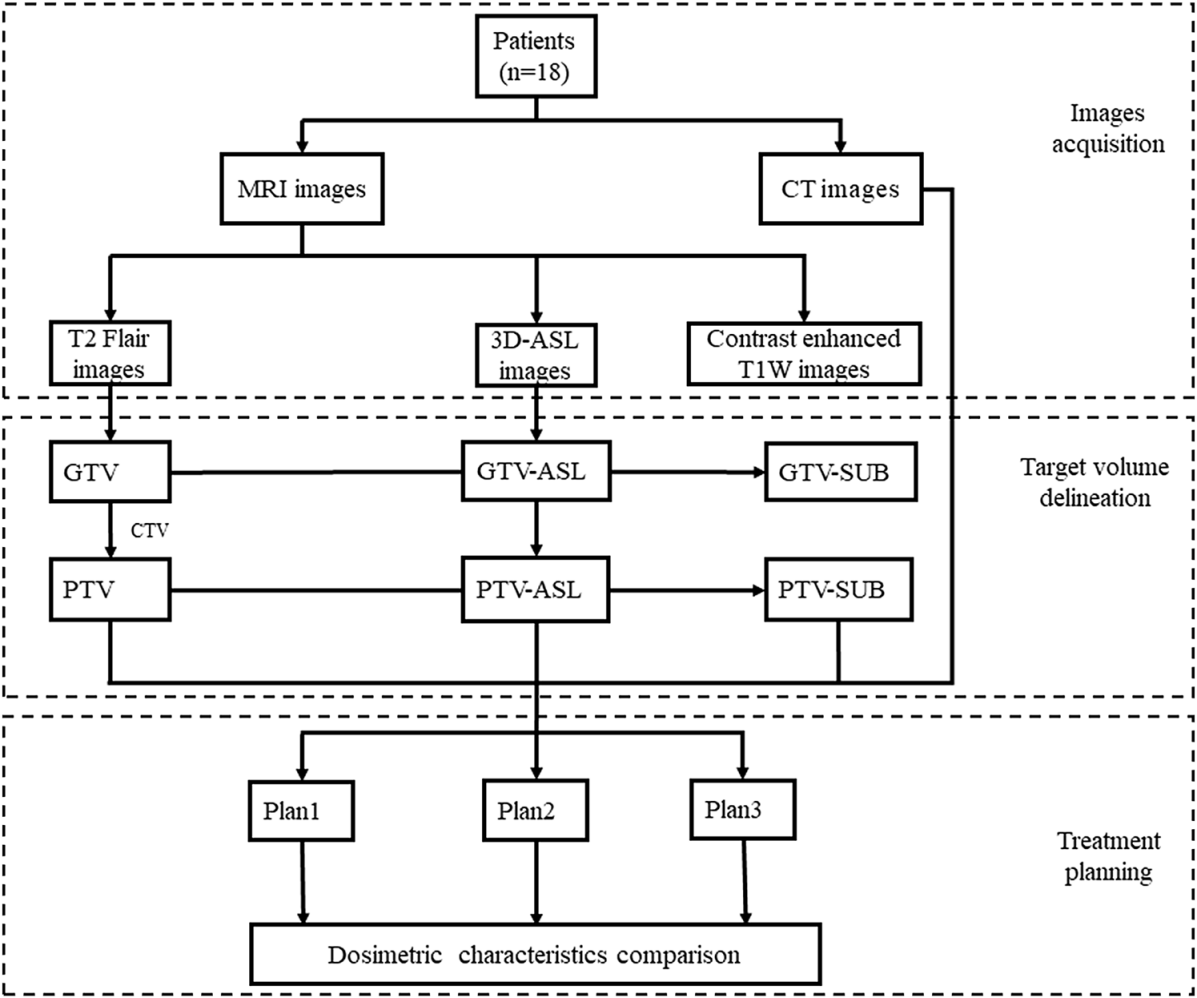
The working flow chart. MRI, magnetic resonance imaging, *CT* computed tomography, *T2 Flair* T2 fluid-attenuated inversion recovery, *3D-ASL* three-dimensional arterial spin labeling, *CE-T1W* contrast-enhanced T1-weighted; *GTV* gross tumor volume, *GTV-ASL* the high-perfusion region on 3D-ASL, *CTV* clinical target volume, *PTV* planning target volume

To compare the three treatment plans, cumulative dose–volume histograms (DVHs) were calculated for all target volumes and OARs for all patients. The D_2%_ and D_98%_ (where D_x_ indicates the dose to x% of the target volume) as well as average dose (D_mean_) were compared for the PTV, PTV-SUB and PTV-ASL. Furthermore, the target coverage, conformity index (CI) and homogeneity index (HI) were calculated. A CI close to one indicates that the shape of the region receiving the reference dose closely matches the shape of the target region, and an HI close to zero indicates good uniformity. The formulas for calculating CI and HI are as follows:

$$ {\text{CI}} = \frac{{\left( {{\text{V}}_{t,ref} } \right)^{2} }}{{{\text{V}}_{t} \cdot {\text{V}}_{ref} }},{\text{HI}} = \frac{{{\text{D}}_{2\% } - {\text{D}}_{98\% } }}{{{\text{D}}_{50\% } }} \cdot $$

where *V*_t_ represents the volume of the target region, *V*_t,ref_ represents the volume of the target region covered by the reference dose, and *V*_ref_ represents the volume of all regions covered by the reference dose. The flow chart of this study is as shown in Fig. 2.

### Statistical analyses

Statistical analyses were performed using SPSS (IBM, version 25.0). Comparisons between two groups were done with paired t tests or Wilcoxon tests, depending on the distribution defined by the Shapiro–Wilk normality test. Comparisons of multiple groups were performed by 2-way analysis of variance (ANOVA), followed by least significant difference (LSD) and Friedman two-way ANOVA, followed by Friedman multiple comparison tests, depending on Levene’s test of variance homogeneity. All data were expressed as mean ± standard deviation, and *P* < 0.05 was considered as a significant difference.

## Results

### Comparison of target volumes

**Fig. 3:**
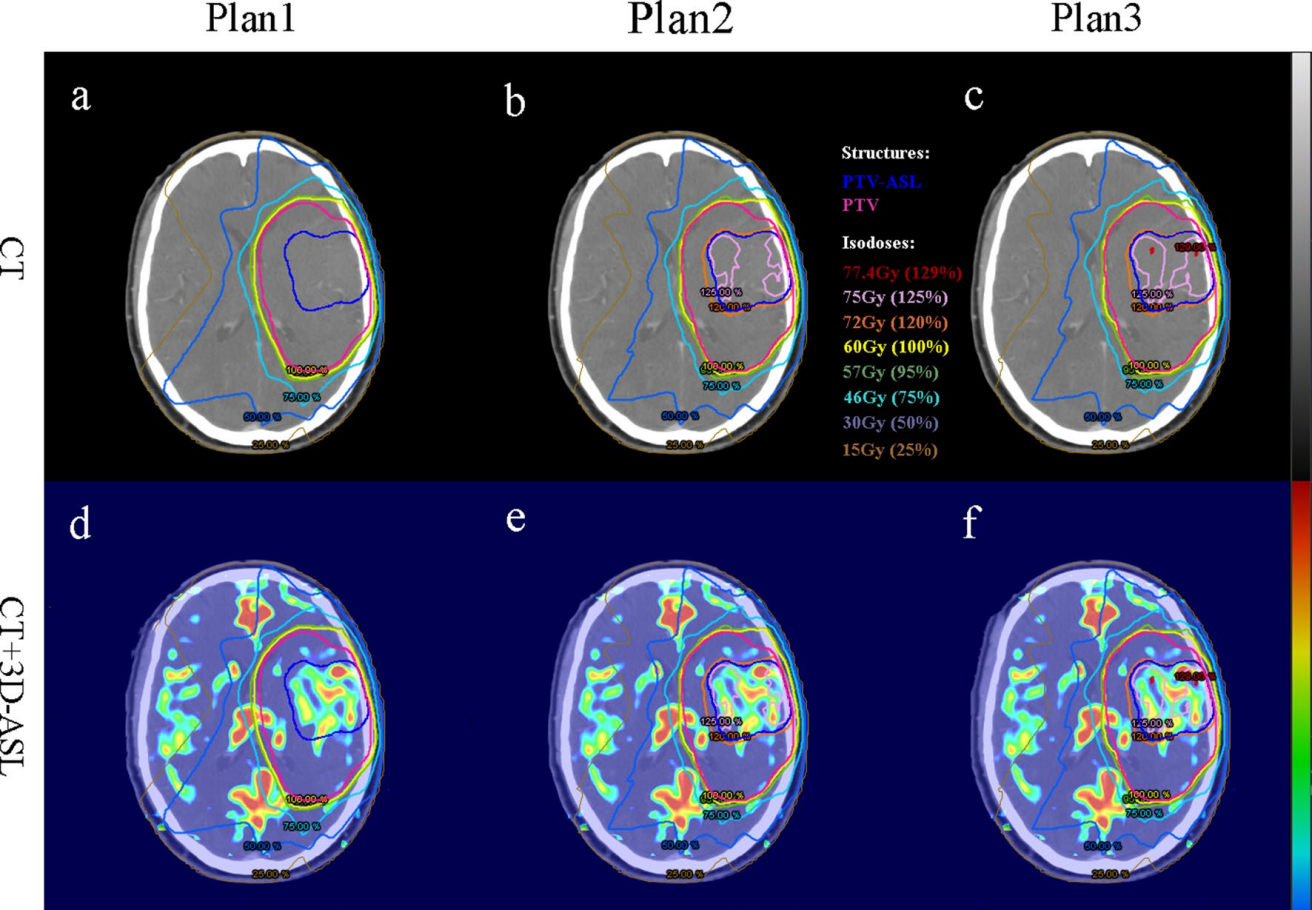
Isodose lines are shown for conventional plan (plan 1) and dose-painted plans (plan 2 and plan 3). Plan 1 (a and d): prescribed a homogeneous dose of 60 Gy to the PTV; plan 2 (b and e) and plan3 (c and f): the dose of PTV-ASL increased by 20% (equivalent to 72 Gy) based on plan 1, without the maximum dose constraint in plan 3. In **a**, **b**, **c** dose distribution is projected on the computed tomography (CT) images while in **d**, **e**, **f** dose distribution is projected on the three-dimensional arterial spin labeling (3D-ASL) images

**Fig. 4:**
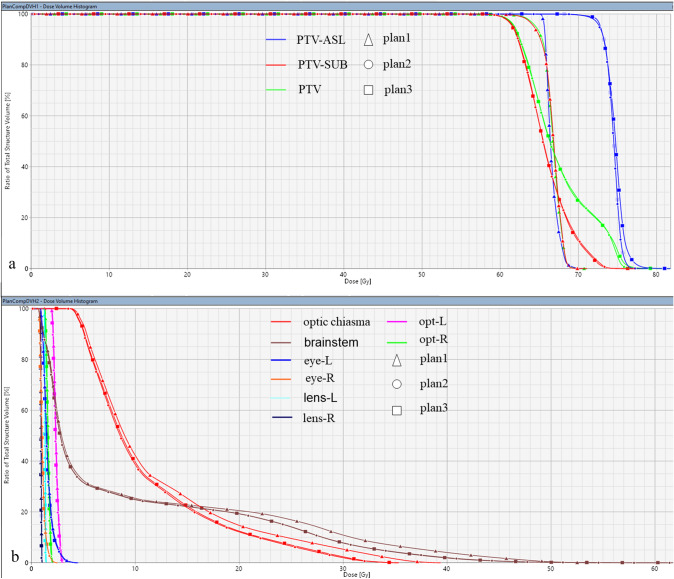
The dose-volume histograms (DVHs) of three treatment plans. The DVHs of the target regions and organs at risks (OARs) are shown in (**a**, **b**) respectively. *PTV* planning target volume

The volumes of the PTV and PTV-ASL were 383.95 ± 255.69 cm^3^ and 90.06 ± 92.03 cm^3^, respectively. The volume ratio of the PTV-ASL to the PTV was (23.49 ± 11.94) % (*Z* = -3.724, *P* = 0.000). Technically, it was feasible to escalate the dose to hyper-perfusion target volume up to 72 Gy without violating dose constraints of normal tissues and, meanwhile, to ensure prescribed doses to the PTVs. Figure 3 shows an example of the dose distribution for the three plans for a sample patient. Figure 4 shows the DVHs of sample target volumes and OARs.

### Dosimetric comparison of target volumes among the three plans

The doses of the PTV, PTV-SUB and PTV-ASL increased in plan 2 and plan 3 than plan 1. The D_2%_ increased by 11.89%, 8.34%, and 14.67% in plan 2 and 15.89%, 8.49%, and 19.84% in plan 3, respectively, with statistically significant differences (*P* < 0.05); the D_98%_ increased by 1.12%, 0.93%, and 16.17% in plan 2 and 0.7%, 0.49%, 15.52% in plan 3, respectively, but only increased significantly for the PTV-ASL in plans 2 and 3 (*P* < 0.05); and D_mean_ increased by 5.15%, 3.60%, and 14.31% in plan 2 and 4.62%, 2.71%, and 14.27% in plan 3, respectively, with statistically significant differences (*P* < 0.05) except for the PTV in plan 3.

**Fig. 5:**
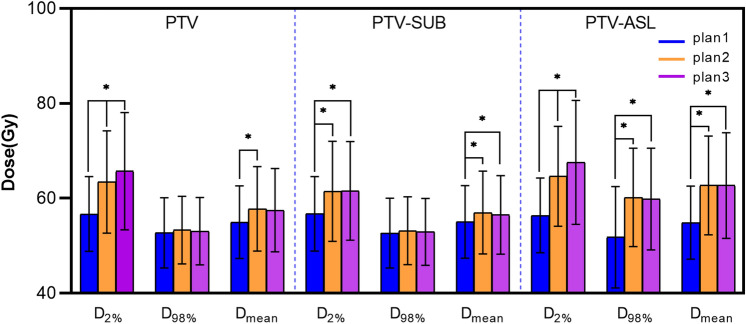
Dosimetric comparison of target volumes among plan 1 (blue), plan 2 (orange) and plan 3 (purple). Compared with plan 1, the dose escalated more significantly in PTV-ASL than PTV-SUB in plan 2 and plan 3. The asterisk (*) indicates *P* < 0.05

Compared with plan 2, the D_2%_ of the PTV, PTV-SUB and PTV-ASL increased by 3.58%, 0.51% and 4.52% in plan 3, respectively, with statistically significant differences for the PTV and PTV-ASL (*P* < 0.05); D_98%_ and D_mean_ decreased by 0.41%, 0.43%, and 0.56% and 0.50%, 0.86%, and 0.03%, respectively, with no statistically significant differences (*P* > 0.05), as shown in Table 3 and Fig. 5.

**Table 3:** The doses of target volumes among the three treatment plans (Gy)

	PTV			PTV-SUB			PTV-ASL		
	D_2%_	D_98%_	D_mean_	D_2%_	D_98%_	D_mean_	D_2%_	D_98%_	D_mean_
Plan1	56.70 ± 7.86	52.72 ± 7.40	54.96 ± 7.65	56.74 ± 7.86	52.67 ± 7.36	55.00 ± 7.65	56.39 ± 7.87	51.81 ± 10.66	54.87 ± 7.69
Plan2	63.44 ± 10.77	53.31 ± 7.12	57.79 ± 8.90	61.47 ± 10.53	53.16 ± 7.15	56.90 ± 8.71	64.66 ± 10.53	60.19 ± 10.37	62.72 ± 10.38
Plan3	65.71 ± 12.36	53.09 ± 7.09	57.50 ± 8.80	61.56 ± 10.41	52.93 ± 7.05	56.50 ± 8.27	67.58 ± 13.05	59.85 ± 10.74	62.70 ± 11.11
Test statistic	31.000	2.563	17.943	27.408	2.111	15.577	31.000	21.444	32.141
*P*	0.000	0.278	0.000	0.000	0.348	0.000	0.000	0.000	0.000
*P*1	0.000	–	0.000	0.000	–	0.000	0.000	0.000	0.001
*P*2	0.000	–	0.200	0.000	–	0.037	0.000	0.000	0.000
*P*3	0.046	–	0.059	0.868	–	0.067	0.046	0.739	0.051

*P1* plan 1 vs. plan 2, *P2* plan 1 vs. plan 3, *P3* plan 2 vs. plan 3

“–”Indicates no data

Test statistic and *P* values were the results of Friedman test

### Plan evaluation

The PTV target coverages for plan 1, plan 2 and plan 3 were comparable (98.45% ± 2.16% vs. 98.70% ± 2.31% vs. 98.20% ± 2.31%, *P* > 0.05). Compared with plan 1, the CIs decreased by 18.60% in plan 2 and 12.79% in plan 3, respectively, with statistically significant differences (*P* < 0.05), while the CIs were comparable between plan 2 and plan 3 (*P* > 0.05). Compared with plan 1, the HIs increased by 1.43 times in plan 2 and 2 times in plan 3, respectively; and increased by 23.53% in plan 3 than in plan 2 (*P* < 0.05).

### Dosimetric comparison of OARs among three plans

Compared to plan 1, the brain stem D_0.1 cc_ and optic chiasma D_max_ were slightly increased by 3.98% and 4.33% in pan 2, and 4.52% and 6.27% in plan 3 respectively, with statistically significant differences (*P* < 0.05), while the absolute doses were safe. The D_max_ of the eyeballs, lens, and optic nerves were comparable among plan 1, plan 2, and plan 3 (*P* > 0.05), as shown in Table 4.

**Table 4:** The doses of OARs among the three treatment plans (Gy)

	Brainstem D_0.1 cc_	Eyeballs D_max_		Lens D_max_		Optic nerves D_max_		Optic chiasma D_max_
		L	R	L	R	L	R	
Plan1	49.95 ± 3.66	19.18 ± 10.34	17.24 ± 9.47	5.11 ± 2.17	5.12 ± 1.89	24.93 ± 16.05	21.96 ± 14.54	46.89 ± 10.70
Plan2	51.94 ± 5.89	20.01 ± 11.30	18.06 ± 9.73	5.99 ± 2.30	5.65 ± 2.09	24.17 ± 16.43	20.79 ± 13.61	48.92 ± 11.71
Plan3	52.21 ± 6.40	19.72 ± 10.99	17.93 ± 9.25	7.19 ± 5.28	6.54 ± 4.20	25.24 ± 17.72	22.08 ± 15.20	49.83 ± 12.27
*F*	5.67	1.531	1.554	2.555	2.098	0.134	0.255	9.357
*P*	0.007	0.231	0.226	0.093	0.138	0.875	0.777	0.001
*P*1	0.101	0.094	0.11	0.348	0.454	0.721	0.563	0.006
*P*2	0.004	0.271	0.176	0.031	0.051	0.887	0.949	0
*P*3	0.713	0.549	0.797	0.202	0.213	0.619	0.522	0.201

*P1* plan1 vs. plan2, *P2* plan1 vs. plan3, *P3* plan2 vs. plan3

*L* left, *R *right, *OARs* organs at risk

*F* and *P* values were the results of 2-way analysis of variance (ANOVA)

## Discussion

Previous study confirmed that MR 3D-ASL images can be used to quantify and analyze tumor perfusion differences in NE-LGGs, and that sub-volume segmentation of the GTV can be performed based on this perfusion difference [21]. In this study, the dose delivered to hyper-perfusion volume derived from 3D-ASL can increased by 10–20% while respecting the constraints to the OAR for NE-LGGs using of SIB technique.

Many LGGs inevitably develop into malignant high-grade gliomas, and even with effective treatment, the survival rate of patients with LGGs remains poor [22, 23]. Radiotherapy can delay the malignant transformation of LGGs, improve the local control rate of tumor, and improve the survival rate and quality of life of patients.

Previously prospective clinical trials, such as EORTC 22,844 (45 vs. 59.4 Gy) and NCCTG 86–72-51 (50.4 vs. 64.8 Gy), showed that high-dose radiotherapy did not improve the survival of patients with LGGs, but rather increased the risk of radiation necrosis and reduced the quality of life [24, 25]. However, Liu et al. [26, 27] demonstrated high-dose radiotherapy (≥ 54 Gy) improved the survival of LGGs, especially for patients with an IDH mutation/1p/19q non-co-deletion or IDH wild-type, while no radio-toxicity was reported in these retrospective studies. Therefore, increasing the radiation dose can improve the survival rates of LGGs patients with special molecular subtypes. And in this study, more than 70% the participants were patients with IDH wild-type LGGs, which may need to be considered for high-dose radiotherapy. High-dose radiotherapy often indicates high local control and efficacy, while synchronously increased the risk of radiation damage to surrounding normal tissues. How to safely and effectively escalate the dose of tumor target volume was the purpose of our study.

NE-LGGs, as a special type of LGGs with significant heterogeneity, require an individualized radiotherapy dose for the tumor target volume. Jakola et al. [28] showed that malignant transformation occurs locally and within the T2WI hyper-intensities in most NE-LGGs patients, and the region of malignant transformation in a new significant contrast enhancement due to the disruptive blood–brain barrier, was much smaller than the edema area. Although coverage of the radiotherapy dose to the tumor can be guaranteed if the edema area is directly defined as the GTV of the NE-LGGs, it is difficult to increase the radiotherapy dose due to the large irradiation range. However, we found that NE-LGGs showed no abnormalities on CE-T1W MRI but showed local high perfusion on 3D-ASL. And all the local hyper-perfusion regions were within edema areas through the fusion images of T2 Flair and 3D-ASL. The study also found that the volume of the hyper-perfusion region (GTV-ASL) was significantly smaller than the volume of the edema area (GTV) by analyzing target volumes. The target volume of dose escalation guided by 3D-ASL was smaller than conventional radiotherapy. Under the same prescription dose, the dose of surrounding healthy tissues is lower. Therefore, it is more conductive to dose escalation ensuring that surrounding healthy tissues were not damaged.

3D-ASL is a functional MRI that does not depend on the destruction of the blood–brain barrier and the paramagnetic contrast agent. It has the advantages of being non-invasive, low cost, simple to operate, and repeatable, and effectively avoids the potential risks caused by an exogenous contrast agent [29]. Related studies have shown that the CBF determined by 3D-ASL is significantly positively correlated with micro-vessel density (*ρ* = 0.567; *P* = 0.029) and vascular endothelial growth factor expression (*r* = 0.604; *P* < 0.001) but significantly negatively correlated with survival (*r* = -0.714, *P* < 0.001) and can be an independent risk factor for OS (HR = 1.028; *P* = 0.010) [14, 30]. More malignant tumor zones tend to have higher tumor blood flow (TBF). Chekhonin et al. [31] have shown that ASL perfusion revealed higher TBF in active tumor growth region compared to peri-focal infiltrative edema zone. Meanwhile, Vallatos et al. [32]also revealed a negative correlation between tumor cell infiltration and perfusion at the tumor margins (*P* = 0.0004) by MRI/histology voxel-to-voxel comparison, and concluded that the quantitative relationship between tumor cell density and perfusion identified surrounding macroscopic tumor could be used to detect marginal tumor cell infiltration with greater accuracy. We defined the high-perfusion region derived from 3D-ASL in GTV as a high-risk region, which was conducted to SIB.

In this study, plan 1 was the conventional plan, and plan 2 and plan 3 were dose-painted plans. Compared with plan 1, the D_2%_, D_98%_ and D_mean_ of the PTV-ASL increased by more than 14% in plan 2 and plan 3; however, these values increased by less than 9% in the PTV-SUB, with D_98%_ increased by less than 1%. This suggested that under the guidance of 3D-ASL, the dose-painted plan can achieve a significant dose boost in hyper-perfused sub-volume region for NE-LGGs while having less impact on the peripheral dose and avoiding dose boost blindness. This is similar to the study of Thureau et al., which was based on 18Flourodeoxyglucose (^18^FDG) PET-CT image-guided dose boost for locally advanced non-small-cell lung cancer [33]. The dose to high recurrence risk areas increased by 12.12% without increasing the radiation dose to OARs, whereas the doses to other areas of the GTV only increased by 2.88%. We also found that the D_2%_ of the PTV, PTV-SUB and PTV-ASL gradually increased in plan 1, plan 2 and plan 3, while the D_98%_ and D_mean_ of each target region were comparable between plan 2 and plan 3. Thus, the elimination of maximum dose constraints is favorable to boost the maximum dose but has no significant effect on the minimum or average dose.

The target coverages of the PTV were comparable among plan 1, plan 2 and plan 3, with the prescribed isodose line wrapping around approximately 95% of the PTV, indicating that all treatment plans for plan 2 and plan 3 met the minimum dose requirements of the target region. Because only the local hyper-perfused regions on 3D-ASL were escalated in plan 2 and plan 3, whereas the edema areas surrounding the hyper-perfused regions received standard doses, the HIs were larger in plan 2 and plan 3 than in plan 1. In this study, the conformality and homogeneity of plan 2 and plan 3 decreased. However, the dose to the tumor target region was significantly increased. Therefore, reducing the homogeneity of the radiation dose is beneficial for dose escalation and protecting OARs.

During radiotherapy for patients with NE-LGGs, radiation damage to surrounding healthy tissues should not be ignored. The dose boost to the tumor target region must be balanced with protecting OARs. The results of this study showed that the dose delivered to OARs was comparable among the three treatment plans, except for brain stem and optic chiasma. However, the absolute doses of all OARs were within a safe range. In addition, the constrained boost plan outperformed the unconstrained plan in terms of OAR protection.

The main limitations of our study were as follows: (1) The number of enrolled patients is relatively small, due to the low incidence of NE-LGGs. Meanwhile, most of patients were operated. Due to the destruction of the blood–brain barrier, the post-operative area was frequently enhanced on enhanced MRI, which cannot be enrolled in the study. (2) Although this is a preclinical technical study, it lays a foundation for future studies on the impact of this technology on the progression free survival (PFS) and OS.

## Conclusion

The dose delivered to hyper-perfusion volume derived from 3D-ASL can increased by 10–20% while respecting the constraints to the OARs for NE-LGGs, which provides a basis for future individualized and precise radiotherapy, especially if the contrast agent cannot be injected or when contrast enhancement is uncertain.

## Abbreviations

LGGs: Non-enhancing low-grade gliomas
CT: Computed tomography
MRI: Magnetic resonance imaging
PET: Positron-emission tomography
DCE: Dynamic contrast-enhanced
OS: Overall survival
T2 Flair: T2 fluid-attenuated inversion recovery
GTV: Gross tumor volume
DSC: Dynamic susceptibility contrast
3D-ASL: Three-dimensional arterial spin labeling
CBF: Cerebral blood flow
MRS: Magnetic resonance spectroscopy
PACS: Picture Archiving and Communication Systems
WHO CNS5: The fifth edition of the WHO Classification of tumors of the central nervous system
NOS: Not otherwise specified
IDH: Isocitrate dehydrogenase
CE-T1W: Contrast-enhanced T1-weighted
EPI: Echo planar imaging
PLD: Post-spin labeling delay
NEX: Number of excitation
TR: Repetition time
TE: Echo time
FOV: Field of view
OARs: Organs at risk
CTV: Clinical target volume
PTV: Planning target volume
SIB: Simultaneous integrated boost
IMRT: Intensity-modulated radiotherapy
DVHs: Dose–volume histograms
D_x_: The dose to x% of the target volume
D_max_: The maximum dose
D_min_: The minimum dose
D_mean_: The average dose
CI: Conformity index
HI: Homogeneity index
ANOVA: Analysis of variance
LSD: Least significant difference
^18^FDG: 18Flourode oxyglucose
